# *Leishmania major*-Infected *Phlebotomus duboscqi* Sand Fly Bites Enhance Mast Cell Degranulation

**DOI:** 10.3390/pathogens12020207

**Published:** 2023-01-28

**Authors:** Laura Sánchez-García, Armando Pérez-Torres, Marco E. Gudiño-Zayas, Jaime Zamora-Chimal, Claudio Meneses, Shaden Kamhawi, Jesus G. Valenzuela, Ingeborg Becker

**Affiliations:** 1 División Ciencias de la Salud, Universidad Autónoma del Estado de Quintana Roo, Chetumal C.P. 77039, Mexico; 2 Departamento de Biología Celular y Tisular, Laboratorio de Inmunología Comparada de Piel y Mucosas, Facultad de Medicina, Universidad Nacional Autónoma de México, Avenida Universidad 3000, Ciudad de México C.P. 04510, Mexico; 3 Unidad de Investigación en Medicina Experimental, Facultad de Medicina, Universidad Nacional Autónoma de México, Avenida Universidad 3000, Ciudad de México C.P. 04510, Mexico; 4 Vector Molecular Biology Section, Laboratory of Malaria and Vector Research, National Institute of Allergy and Infectious Diseases, National Institutes of Health, Rockville, MD 20852, USA

**Keywords:** mast cells, Leishmania, sand fly, TNF-α, tryptase, inflammation, neutrophils

## Abstract

*Leishmania* parasites infect mammalian hosts through the bites of sand fly vectors. The response by mast cells (MC) to the parasite and vector-derived factors, delivered by sand fly bites, has not been characterized. We analyzed MC numbers and their mediators in BALB/c mice naturally infected in the ear with *Leishmania major* through the bite of the sand fly vector *Phlebotomus duboscqi* and compared them to non-infected sand fly bites. MC were found at the bite sites of infective and non-infected sand flies throughout 48 h, showing the release of granules with intense TNF-α, histamine, and tryptase staining. At 30 min and 48 h, the MC numbers were significantly higher (*p* < 0.001) in infected as compared to non-infected bites or controls. Neutrophil recruitment was intense during the first 6 h in the skin of infected and non-infected sand fly bites and decreased thereafter. An influx of neutrophils also occurred in lymph nodes, where a strong TNF-α stain was observed in mononuclear cells. Our data show that MC orchestrate an early inflammatory response after infected and non-infected sand fly bites, leading to neutrophilic recruitment, which potentially provides a safe passage for the parasite within the mammalian host.

## 1. Introduction

Mast cells (MC) play a main role during inflammation after infection or tissue injury. The strategic location of MC in the skin, as well as in the intestinal and airway mucosae, enable them to orchestrate the early inflammatory response and to regulate adaptive immunity [1,2,3,4,5,6]. MC are activated by diverse stimuli, including complement components, pathogens, toxins, and immunoglobulin-binding proteins [7], leading to degranulation and release of highly diverse mediators stored in their cytoplasmic granules, which contribute to vascular permeability, adhesiveness, diapedesis, and transmigration of circulating leukocytes into the surrounding tissues [4,7,8]. MC enhance the inflammatory process through paracrine regulation, where within minutes of degranulation, they activate other cell types leading to intense inflammation [9,10]. The inflammatory environment created by MC after detecting a pathogen enables immune cells to travel to infection sites, as well as to draining lymph nodes [11].

In Leishmania infections, MC have been shown to orchestrate the immune response, amplifying the functionality of dendritic cells and aiding CD8 T cell priming [12,13,14]. Furthermore, MC recruit dendritic cells that regulate adaptive immune responses in L. major infections [12,13,14]. In vitro studies of MC incubated with different Leishmania species or parasite antigens have shown that MC release TNF-α, β-hexosaminidase, tryptase and IL-4 [15]. The crucial role of MC in promoting Leishmania infections has also been shown in mouse models, where their inactivation prior to L. major infection of BALB/c or other mice strains reduced the infection [16]. Similarly, MC of susceptible mouse models infected with *Leishmania mexicana* showed an earlier activation response, as compared to resistant mice [17]. Recently, a study of the increased severity of visceral leishmaniasis after vector-transmission of *L. donovani* showed that MC degranulation is significantly enhanced 3 h after infected bites [18]. Together, these data suggest that the early inflammatory signals released by MC favor parasite dissemination and the establishment of the infection. 

During natural *Leishmania* infection by vector bites, MC are exposed to a complex infectious inoculum containing several vector-derived factors of sand fly and *Leishmania* origin. These include the promastigote secretory gel, parasite exosomes, gut microbiota, and vector saliva, all of which have been shown to modulate the host immune response [18,19,20]. These vector-derived factors are released during the sand fly bite and can contribute to the inflammatory response with long-lasting effects on the host [21,22,23]. Since the response of MC to the combined stimuli exerted by the parasite and vector-derived factors has not been thoroughly characterized, we analyzed the MC response in mice naturally infected with *L. major* through the bites of the sand fly vector *Phlebotomus (Ph.) duboscqi*. Using immunohistochemistry, we describe the infiltration of MCs and their release of inflammatory mediators in the skin during the first 48 h after sand fly bites. Furthermore, we analyze the kinetics of neutrophil infiltration in the skin and draining lymph nodes at 30 min, 6 h, 24 h, and 48 h after the bite of *L. major*-infected and non-infected sand flies. 

## 2. Materials and Methods

### 2.1. Mice

Female BALB/c mice aged 6 to 8 weeks (Charles River Laboratories Inc. Wilmington, Delaware DE) were used. The mice were maintained under pathogen free conditions at NIAID animal facilities, following the guidelines established by the NIAID Institutional Animal Care and Use Committee.

### 2.2. Sand Flies 

The methods described in the literature [23,24] were followed with some modifications. *Ph. duboscqi* female sand flies (NIH colony) were maintained at the Laboratory of Malaria and Vector Research at the National Institute of Allergy and Infectious Diseases. Three to four days after eclosion, the sand flies were fed on a 20% sucrose solution until needed.

### 2.3. Leishmania major Culture

The methods described in the literature [24] were followed with some modifications. *L. major* (strain SD 75.1) promastigotes were cultured in Schneider’s insect medium (Sigma-Aldrich, St. Louis, MO, USA) containing 10% fetal bovine serum (Invitrogen, Carlsbad, CA, USA), 100 units/mL penicillin, 100 ug/mL streptomycin, and 0.292 mg/mL glutamine (Invitrogen, Carlsbad, CA, USA) at 25 °C. Procyclic promastigotes were washed three times in PBS, centrifuged at 179× *g* during 10 min at room temperature (RT), counted, and added to rabbit blood at a final concentration of 2 × 10^6^ parasites/mL. 

### 2.4. Sand Fly Infection 

The methods described by Teixeira et al. 2014 were followed with some modifications [24]. Five-day-old *Ph. duboscqi* females were infected by feeding through a chicken-skin membrane that was fixed to a glass feeder containing 2 × 10^6^ *L. major* promastigotes and 30 μL penicillin/streptomycin (10,000 units penicillin/10 mg streptomycin) per mL of defibrinated rabbit blood. Engorged sand flies were separated and maintained on 70% relative humidity, 25–26 °C and photoperiods of 14:10 h of light:dark. The sand flies were dissected at 2, 6, and 10 days after the infective feed to estimate the infection intensity and maturity. 

The parasite load and percentage of metacyclics per midgut in *L. major*-infected *P. duboscqi* sand flies were determined on the day of transmission. The infection status was scored based on the total parasite load and percentage of metacyclic promastigotes found in the midguts of the sand flies. Generally, counts with 60–80% metacyclics were considered excellent transmissible infections, those 40–50% metacyclics as moderately transmissible infections, and below 40% as poor infections. Metacyclic promastigotes were distinguished by their morphology and motility. Metacyclic promastigotes were distinguished by their morphology and motility. They have a small cell body size relative to the length of their flagellum and have a unique vigorous motility [25,26,27].

Depending on sand fly availability, 5–10 flies were anesthetized with CO_2_, washed in 5% soap solution, and rinsed in PBS prior to dissection. Each midgut was macerated in an Eppendorf tube containing 50 µL of PBS using a pestle (Kimble Chase, Vineland, NJ, USA). Based on morphology and movement, the parasite load and the percent metacyclics per midgut were determined under a compound microscope using a hemocytometer. Only sand flies harboring mature transmissible infections in ≥60% of counted specimens were allowed to feed on anesthetized BALB/c mice 10–12 days post infection.

### 2.5. Infection of Mouse Ears by Sand Flies 

Six mice were anesthetized intraperitoneally with ketamine (100 mg/kg) mixed with xylazine (10 mg/kg). Ophthalmic ointment (Major Pharmaceuticals) was topically applied to the eyes to prevent corneal dryness. Three mice were exposed to ten uninfected sand flies and 3 mice were exposed to ten sand flies harboring mature infections. The sand flies were applied to both ears of each mouse using vials with a meshed surface held in place by custom-made clamps. Sham mice exposed to empty vials were used as negative controls. Sand flies were allowed to feed for 2–3 h in the dark. After exposure to mice, the sand flies were aspirated from vials, anesthetized with CO_2_, washed in 5% soap solution, rinsed, and placed in a petri dish containing PBS. The number of blood-fed flies was counted under a stereomicroscope. Mice were euthanized 30 min, 6 h, 24 h, and 48 h after sand fly bites. For every experiment, both earlobes of 3 mice were used. Each earlobe was cut into 3 segments, and 2 replicates were made for every experimental condition (n = 36 earlobe segments analyzed for every condition). 

### 2.6. Scoring of Sand Fly Infection Status Post-Transmission

After scoring of blood feeding status, sand flies in each vial applied to an ear were analyzed separately to assess the infection status post-exposure to mice. Each sand fly was dissected and observed under a compound microscope for density and location of midgut parasites and for the presence of motile metacyclics. Based on these parameters, the number of sand flies harboring excellent (high metacyclic density) or moderate (moderate metacyclic density) transmissible infections were determined. 

### 2.7. Histology and Immunohistochemistry of Ears and Lymph Nodes after Bites of L. major-Infected and Non-Infected Sand Flies 

The ears exposed to sand fly bites were cut into 3 segments along the maximum number of bite sites (that are distinct upon examination of the ears), and multiple step sections were obtained to ensure that several bite sites were included. The two ears of exposed mice were gently flattened onto a piece of thick paper to avoid curling and cut by scissors into three equally sized fragments (0.7 × 1.6 cm). They were fixed in 4% paraformaldehyde in 0.1 M Tris-HCl buffer (pH 7.2) for 24 h. Mandibular draining lymph nodes were fixed in same manner, and all specimens were paraffin-embedded to obtain 4 μm thick tissue sections, which were mounted on positively charged slides (Superfrost plus^®^; Shandon Inc., Pittsburgh, PA, USA). All tissue sections were stained with hematoxylin–eosin (H&E) or 2% toluidine blue (Sigma Aldrich, St. Louis, MO, USA) for histopathological analysis and MC identification, respectively. MC identification was done following the metacromatic principle [28].

The number of MC and neutrophils were counted in the three histological sections per earlobe using a calibrated 40 × objective. The total number of each cell type was reported per mm^2^. Mandibular draining lymph nodes were analyzed using serial tissue sections stained with H&E. All slides were analyzed two times by two independent investigators. 

The staining of TNF-α, histamine, and tryptase was assessed in paraffin-embedded ear tissue sections by immunohistochemistry, whereas in mandibular draining lymph nodes, only TNF-α was analyzed. Briefly, tissue sections were de-waxed with xylene, rehydrated with 0.1 M Tris-HCl buffer (pH 7.2), and transferred to plastic Coplin jars containing 0.1 M citrate buffer (pH 6.0) for antigen retrieval. Slides in the Coplin jar were heated in a pressure cooker for 20 min at 200 °C, followed by 10 min at 100 °C. The slides were cooled in the jar at (RT) for 15 min and then transferred to 0.1 M Tris-HCl buffer (pH 7.2) until needed. After antigen retrieval, endogenous peroxidase was inhibited by incubation for 30 min at RT with 3% hydrogen peroxide diluted in methanol. Slides were then incubated for 1 h at RT in a solution containing 0.1 M Tris-HCl buffer (pH 7.2), 2% BSA, and 0.01% Triton X-100 to reduce nonspecific background staining. Slides were incubated overnight at 4 °C with specific primary anti-TNF-α (1:50 anti-mouse N-19 sc 1350 Santa Cruz), anti-tryptase (1:100 Mast Cell Tryptase; anti-rabbit FL-275-Santa Cruz Biotech), and anti-histamine (1:100 anti-rabbit ab78335 Abcam) antibodies diluted in 0.1% BSA/0.1 M Tris-HCl buffer (pH 7.2). After three washes in the same buffer, slides were incubated for 30 min with biotinylated secondary antibodies anti-mouse IgG (diluted 1:50) (Jackson Immuno Research Laboratories cat # 115035003) or with anti-rabbit IgG (diluted 1:50) (Sigma Aldrich, St. Louis, MO, USA) for 1 h at RT. The avidin–biotin–HRP complex was used, and the reaction was developed with hydrogen peroxide and 3,3′-diaminobenzidine, according to the supplier’s instructions (Biocare Medical, Pacheco, CA, USA). Finally, tissue sections were counter-stained with hematoxylin (Sigma HHS16) for 1 min. Only dark brown-colored cells with visible nucleus and cytological features of MC, and neutrophils were identified as positively stained cells. 

### 2.8. Statistical Analysis

MC and neutrophil counts were analyzed using repeated measures ANOVA running Tukey’s test. The statistical analysis was done using Graph Pad Prism 7.0 (Dotmatics, San Diego, CA, USA). 

## 3. Results

### 3.1. Post-Exposure Status of L. major-Infected and Non-Infected Phlebotomus Duboscqi Sand Flies

**Figure 1:**
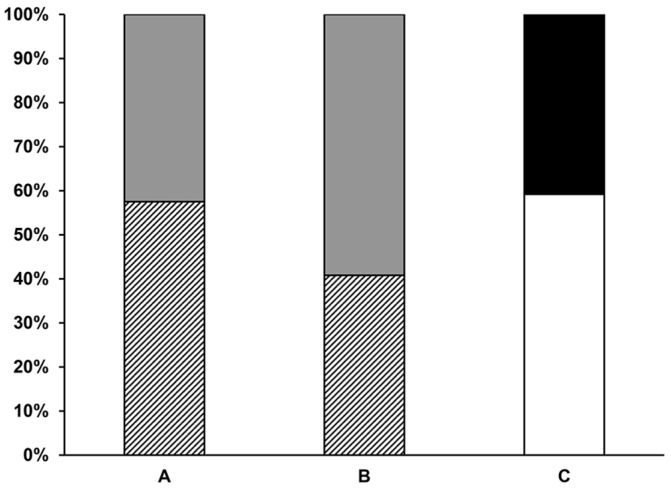
Post-sand fly exposure feeding score and infection status of *Phlebotomus duboscqi.* (**A**) Post-exposure feeding score of 10 non-infected sand flies. (**B**) Post-transmission feeding score of 10 infected sand flies. (**C**) Post-transmission *L. major* infection status in 10 infected sand flies. Striped bars, fully fed; Grey bars, partially fed; White bars, the number of sand flies showing excellent transmissible infections; Black bars, number of sand flies showing moderate transmissible infections. Data are representative of two independent experiments; n = 6 ears per group.

The blood feeding score of the 10 sand flies per ear applied to ears of individual mice was analyzed for both non-infected (Figure 1A) and infected sand flies (Figure 1B). For infected sand flies, the number of specimens harboring transmissible infections post-exposure were also determined (Figure 1C). Infected sand flies showed a low percent of fully fed (40.8%), as compared to partially fed, sandflies (59.2%) (Figure 1A,B). All the sand flies showed either excellent (59.2%) or moderate (40.8%) transmissible infections, containing mobile metacyclic parasites (Figure 1C). 

### 3.2. Mast Cell Response to Sand Fly Bites

#### 3.2.1. Skin Mast Cell Numbers after Infected and Non-Infected Sand Fly Bites

**Figure 2:**
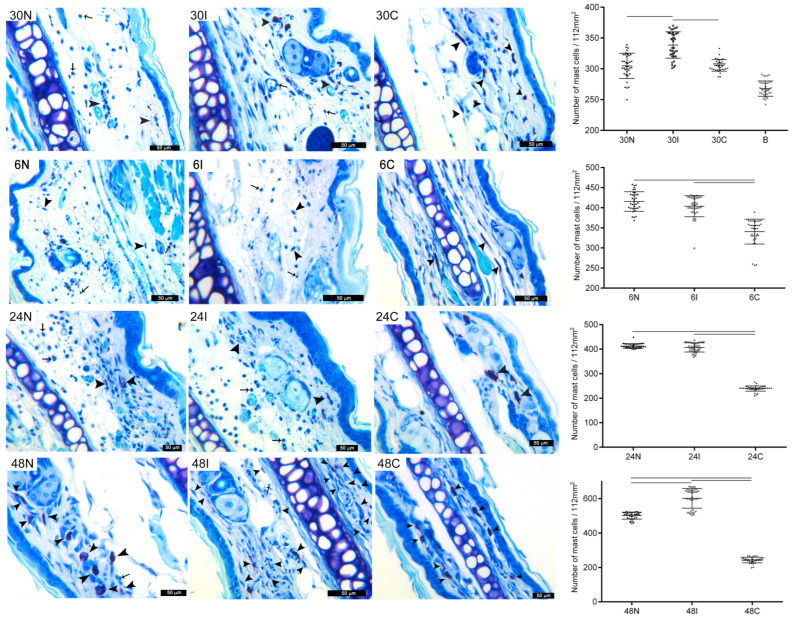
Mast cell numbers in mouse tissues after sand fly bites. Mast cell (MC) numbers in tissues after non-infected bites at 30 min, 6 h, 24 h, and 48 h (30N, 6N, 24N, and 48N); *Leishmania*-infected sand fly bites (30I, 6I, 24I, and 48I); or in controls exposed to an empty vial (30C, 6C, 24C, and 48C). B, basal number of MC in unexposed ears. Counts of MC numbers were made in 112mm^2^. Black arrowheads: MC identified by toluidine blue staining as large cells containing numerous deep violet metachromatic granules of various sizes; black arrows: neutrophils identified by their tri-lobular nuclei; Scale = 50 μm. Black cubes in graphs represent non-infected sand flies, white cubes represent infected sand flies and black dots represent controls. Bars indicate significant differences (*p* < 0.001) between groups. Cumulative data (n = 12 ears with 36 ear sections) and representative images of each experimental condition from two independent experiments are shown. Data are presented as mean ± SD.

Thirty minutes after sand fly bites, an increase in MC numbers in the ear of mice is observed, reaching higher levels in ears exposed to infected sand fly bites (30I, 334 ± 20), as compared to non-infected sand fly bites (30N, 304 ± 21) (Figure 2). It is worthy to note that MC numbers in controls exposed to empty vials (30C) were similar to those of non-infected bites (30N, 305 ± 9). MC numbers in controls reached near-basal levels at 24 h (24C, 239 ± 11) (Figure 2). In contrast, the numbers of MC in tissues exposed to infected or non-sand fly bites continued to increase at comparable levels during the next 24 h. Thus, after 6 h, MC numbers in infected (6I, 404 ± 19) and non-infected sand flies (6N, 411 ± 23) reached comparable levels, which remained unaltered during 24 h (Figure 2). Yet, at 48 h, a new surge in MC numbers was evidenced in both infected and non-infected bites. However, at this time point, infected sand fly bites (48I, 601 ± 56) elicited significantly higher (*p* < 0.001) numbers of MC, as compared no non-infected bites (48N, 502.37 ± 19). Figure 2 depicts images of mast cells at the different timepoints in each group. A concomitant infiltration of neutrophils is also evidenced during the different time points. 

#### 3.2.2. Skin MC Degranulation after Infected and Non-Infected Sand Fly Bites

**Figure 3:**
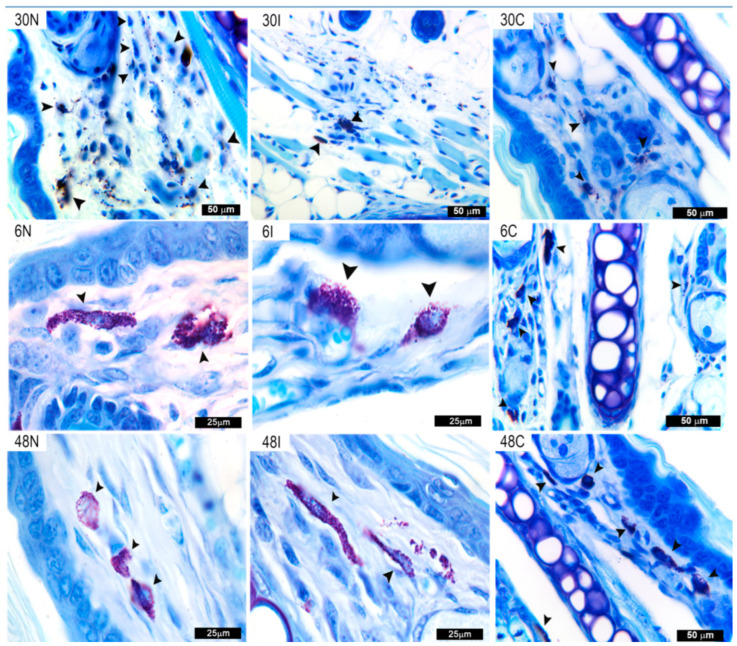
MC degranulation after Ph. duboscqi bites. Mast cell degranulation after non-infected sand fly bites at 30 min, 6 h, and 48 h (30N, 6N, and 48N), *Leishmania major*-infected sand fly bites (30I, 6I, 48I) or in controls exposed to an empty vial (30C, 6C, and 48C). MC (black arrowheads) are defined by numerous cytoplasmic deep violet metachromatic granules of various sizes. Bar = 50 μm in 30N, 30I, 30C, 6C, and 48C. Bar = 25 μm in 6N, 6I, 48N, and 48I. Representative images of each experimental condition from two independent experiments are shown; n = 4.

Toluidine blue staining showed that MC release abundant metachromatic granules as early as 30 min after *L. major*-infected and non-infected sand fly bites, as compared to controls where granules remain contained within MC (Figure 3). After having released abundant granules at 30 min, an accumulation of granules within the cytoplasm was again observed in some MC at 6 h after non-infected and infected sand fly bites, some of which were in the process of being released. It is not clear whether new MC are recruited from surrounding tissues after 6 h, or if the granules within MC represent a possible “de novo” synthesis. After 48 h, most of the granules are still found within MC in both infected and non-infected *Ph. duboscqi* bites. MC from controls exposed to empty vials showed orderly aggregated granules within the cells (Figure 3).

#### 3.2.3. Skin MC Degranulation Leads to Neutrophil Infiltration 

**Figure 4:**
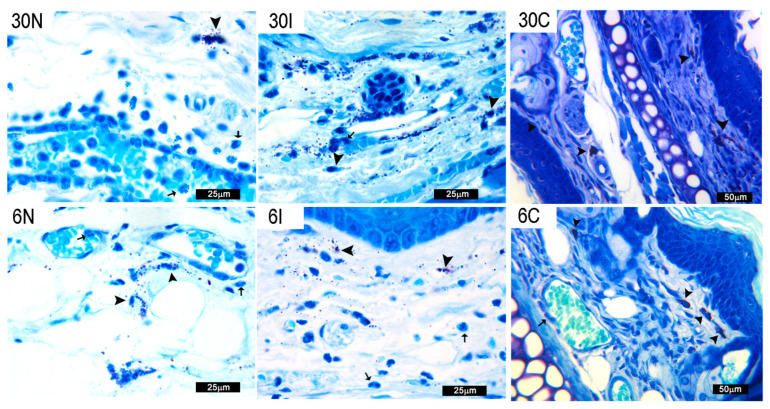
Mast cell degranulation with neutrophil infiltration. Neutrophil infiltration at sites of mast cell degranulation at 30 min (30N and 30I) and 6 h (6N and 6I) after non-infected and infected sand fly bites or in controls not exposed to sand fly bites or parasites at 30 min and 6 h (30C and 6C). Black arrowheads: MC identified by toluidine blue staining as large cells containing numerous deep violet metachromatic granules of various sizes. Black arrows: neutrophils identified by their tri-lobular nuclei. Bars = 25 μm in 30N, 30I, 6N, and 6I; Bars = 50 μm in 30C and 6C. Representative images of each experimental condition from two independent experiments are shown; n = 4.

MC degranulation correlates with a rapid diapedesis of neutrophils. Neutrophil extravasation was evidenced in capillaries close to MC granules after 30 min and 6 h of *L. major*-infected and non-infected sand fly bites (Figure 4). Controls also showed neutrophils close to vessel walls and initiating diapedesis.

#### 3.2.4. MC and Neutrophils Show TNF-α Staining after *Ph. duboscqi* Bites

**Figure 5:**
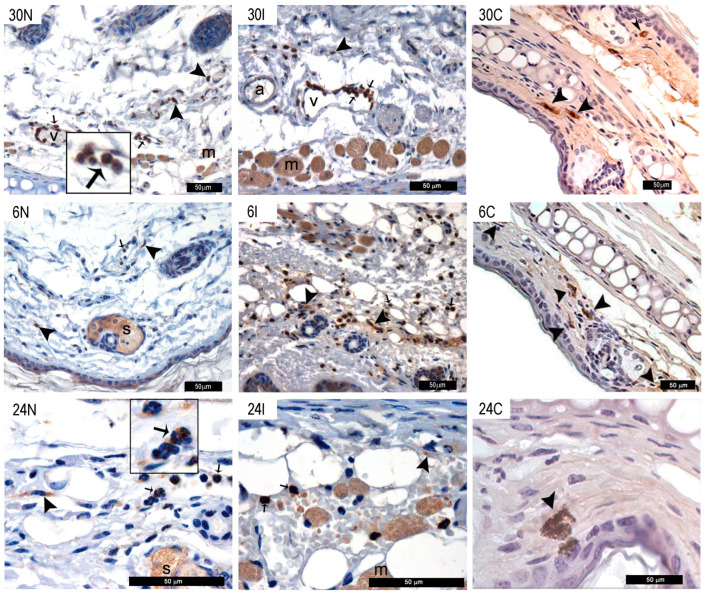
TNF-α staining after *Ph. duboscqi* bites. TNF-α staining in MC and neutrophils after non-infected bites at 30 min, 6 h, and 24 h (30N, 6N, and 24N) and *Leishmania major*-infected sand fly bites (30I, 6I, and 24I) or in controls not exposed to sand fly bites or parasites (30C, 6C, and 24C). TNF-α is evidenced by brown staining (peroxidase) in MC (black arrowheads), neutrophils (black arrows), sebocytes (s), and muscle tissue (m). Venules (v); Arteries (a). Enlarged insets of images 30N and 24N show brown peroxidase TNF-α staining in neutrophils with the typical tri-lobulated nuclei. Bars = 50 μm Representative images of each experimental condition from two independent experiments are shown; n = 4.

In an attempt to establish whether the rapid degranulation observed in MC after *Ph. duboscqi* bites would result in the release of proinflammatory molecules, we analyzed TNF-α, histamine and tryptase staining in ear tissues at different timepoints after sand fly bites by immunohistochemistry. TNF-α was evidenced in dermal MC and recruited neutrophils after 30 min to 24 h in infected and non-infected sand fly bites (Figure 5), (Supplementary Figure S1). Neutrophil diapedesis was observed in venules at 30 min after infected sand fly bites (Figure 5). Arteries showed no TNF-α stained cells. In animals not exposed to sand fly bites or parasites, TNF-α was only found in MC and not in neutrophils or other cell types at all timepoints investigated (Figure 5).

#### 3.2.5. MC Show Histamine Staining after *Ph. duboscqi* Bites

**Figure 6:**
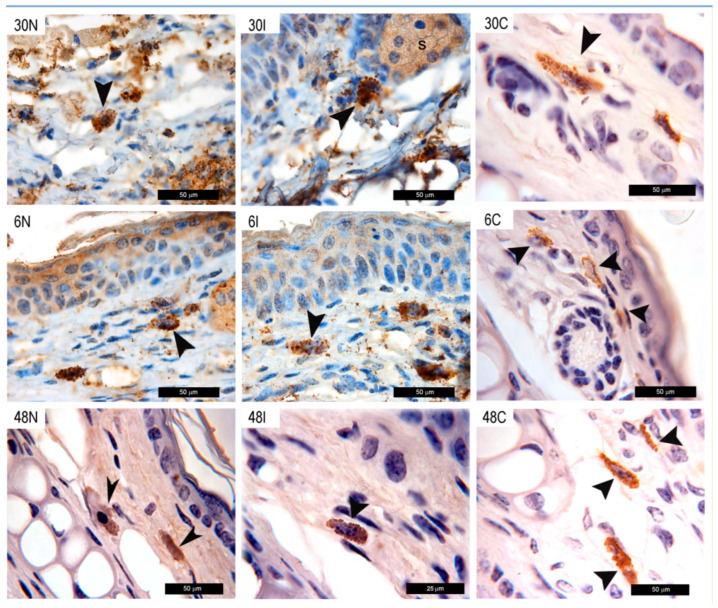
Histamine staining following *Ph. duboscqi* bites. Histamine staining after non-infected bites at 30 min, 6 h, and 48 h (30N, 6N, and 48N); *Leishmania*-infected sand fly bites (30I, 6I, and 48I); or in controls not exposed to sand fly bites or parasites (30C, 6C, and 48C). Histamine is evidenced by brown staining (peroxidase) in MC (black arrowheads). Bars = 50 μm in 30N, 30I, 30C, 6N, 6I, 6C, 48N, and 48C. Bars = 25 μm in 48I. Representative images of each experimental condition from two independent experiments are shown; n = 4.

In addition to TNF-α, an intense histamine staining permeated throughout the ear tissue between 30 min and 48 h after infected and non-infected sand fly bites, as compared to controls not exposed to sand fly bites or parasites, where histamine staining was restricted to the cytoplasm of MC (Figure 6). The distribution of the stain after bites suggests a histamine burst, since histamine granules were distributed as massive bulks at 30 min. After 6 h, a renewed presence of different sized granules could be observed in the MC cell cytoplasm, suggesting different maturation stages of the granules. At 48 h, an attenuated release of histamine was observed in non-infected and infected sand fly bites. In animals not exposed to sand fly bites or parasites, histamine was only found inside MC at all timepoints investigated (Figure 6).

#### 3.2.6. MC Show Tryptase Staining after *Ph. duboscqi* Bites

**Figure 7:**
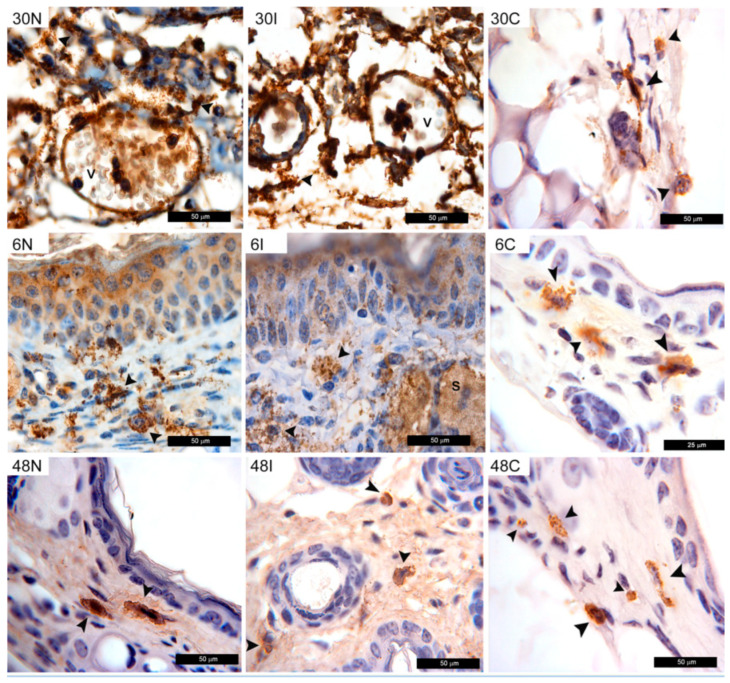
Tryptase staining following *Ph. duboscqi* bites. Tryptase staining after non-infected bites at 30 min, 6 h, and 48 h (30N, 6N, and 48N); *Leishmania*-infected sand fly bites (30I, 6I, and 48I); or in controls not exposed to sand fly bites or parasites (30C, 6C, and 48C). Tryptase is evidenced by brown staining (peroxidase) in MC (black arrowheads), venules (v), and sebocytes (S). Bars = 50 μm in 30N, 30I, 30C, 6N, 6I, 48N, 48I, and 48C. Bars = 25 μm in 6C. Representative images of each experimental condition from two independent experiments are shown; n = 4.

There was also a strong tryptase staining in MC granules, which were spread abundantly over the tissue at every time point beginning at 30 min and continuing through 6 and 48 h after sand fly bites (Figure 7). After 30 min, a massive tryptase staining was observed in MC after infected and non-infected *Ph. duboscqi* bites, which was not present in controls. A strong tryptase mark was also observed on endothelial cells of venules. After 6 h, tryptase was found throughout the tissues and a strong stain was found in the granules inside MC. At 48 h, tryptase staining was mostly found in MC, which showed comparable staining after bites of infected or non-infected sand flies. In control animals not exposed to sand fly bites or parasites, tryptase was only found inside MC at all timepoints investigated (Figure 7). 

### 3.3. Neutrophil Count in Tissues of Non-Infected and L. major-Infected Ph. duboscqi Sand Flies

**Figure 8:**
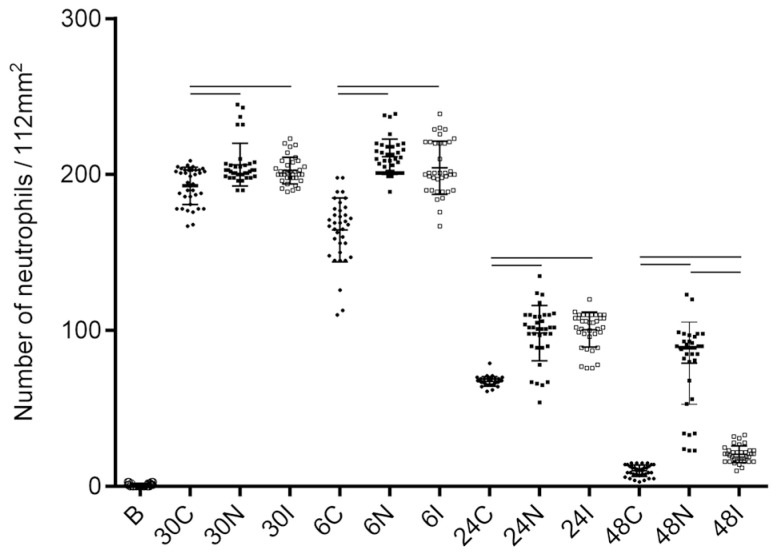
Neutrophil counts in BALB/c earlobes following *Ph. duboscqi* bites. The number of neutrophils in tissues after non-infected bites at 30 min, 6 h, 24 h, and 48 h (30N, 6N, 24N, and 48N); *Leishmania*-infected sand fly bites (30I, 6I, 24I, and 48I); or in controls exposed to an empty vial (30C, 6C, 24C, and 48C). B, basal number of neutrophils in unexposed ears. Bars indicate significant differences (*p* < 0.001) within each group. For every experimental condition, both earlobes of 3 mice were used. Each earlobe was cut into 3 segments and 2 replicates were made (n = 36 earlobe segments were counted for every condition).

An intense neutrophilic infiltration was observed between 30 min and throughout 6 h in tissues after sand fly bites (Figure 8). At 30 min, control tissues exposed to empty vials also showed neutrophilia, although to a lower degree. This initial neutrophilia in controls was transient and began to diminish at 6 h, reaching near-basal levels within 48 h. At all the time points throughout the study, the transient non-specific neutrophil response in controls was always significantly lower, as compared to tissues exposed to sand fly bites. In contrast, neutrophils in tissues exposed to sand fly bites remained elevated during the first 6 h and gradually decreased after 24 h, reaching similar levels in tissues exposed to infected and non-infected sand fly bites. After 48 h, the number of neutrophils in tissues subjected to non-infected sand fly bites remained constant, whereas they diminished significantly after infected bites (Figure 8). 

### 3.4. Neutrophil Infiltration into Tissues after Infected and Non-Infected Sand Fly Bites

**Figure 9:**
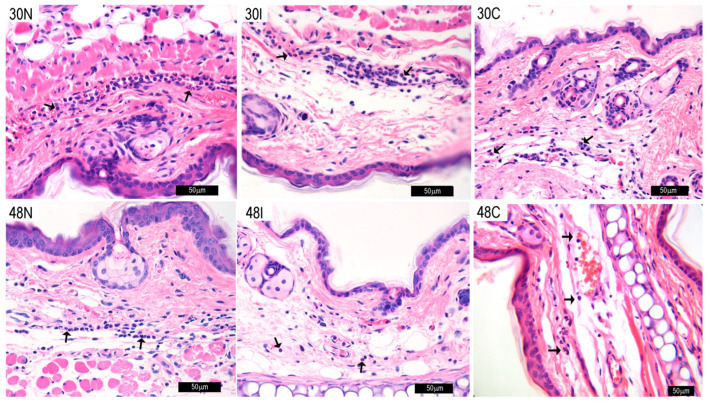
Neutrophil infiltration in earlobes following Ph. duboscqi bites. Vascular congestion, vasodilatation and diapedesis of neutrophils into tissues at 30 min after non-infected (30N), infected sand fly bites (30I), and in controls (30C). Neutrophil diapedesis and tissue localization in non-infected (48N), infected (48I) sand fly bites, and in controls (48C) at 48 h. Neutrophils can be identified by their tri-lobular nuclei. Black arrows: neutrophils. Bars = 50 μm. Representative images of each experimental condition from two independent experiments are shown; n = 4.

The intense neutrophil infiltration in tissues exposed to infected and non-infected sand fly bites was also evidenced in the blood vessels at 30 min, showing a severe vascular congestion, as well as initial diapedesis into the tissues that was absent in the blood vessels of controls (Figure 9). At 48 h, neutrophils were mostly in the process of diapedesis or already distributed within the tissues and was more intense in non-infected as compared to infected sand fly bites.

### 3.5. Lymph Node Activation after Sand Fly Bites

#### 3.5.1. Neutrophil Influx into Lymph Nodes after Sand Fly Bites

**Figure 10:**
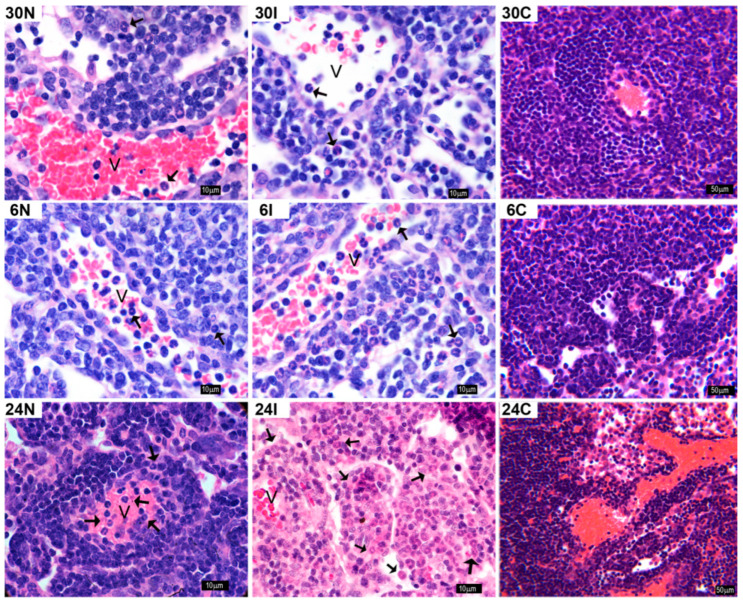
Neutrophil infiltration in the mandibular lymph node after *Ph. duboscqi* bites. Neutrophil infiltration in the mandibular lymph node after non-infected at 30 min, 6 h, and 24 h (30N, 6N, and 24N); *Leishmania major*-infected sand fly bites (30I, 6I, and 24I); or in controls not exposed to sand fly bites or parasites (30C, 6C, and 24C). Some neutrophils are evidenced within venules (v). Neutrophils can be identified by their tri-lobular nuclei. Black arrows: neutrophils. Bars = 10 μm for 30N, 30I, 6N, 6I, 24N, and 24I. Bars = 50 μm in 30C, 6C, and 24C. Representative images of each experimental condition from two independent experiments are shown; n = 4.

H&E staining showed an influx of neutrophils into the lymph node after 30 min, 6 and 24 h in both infected and non-infected sand-fly bites (Figure 10). Neutrophils enter the lymph nodes through high endothelial venules (HEVs) to reach the paracortex and medullary cords. 

#### 3.5.2. Lymph Node Activation Shows TNF-α Release after Sand Fly Bite

**Figure 11:**
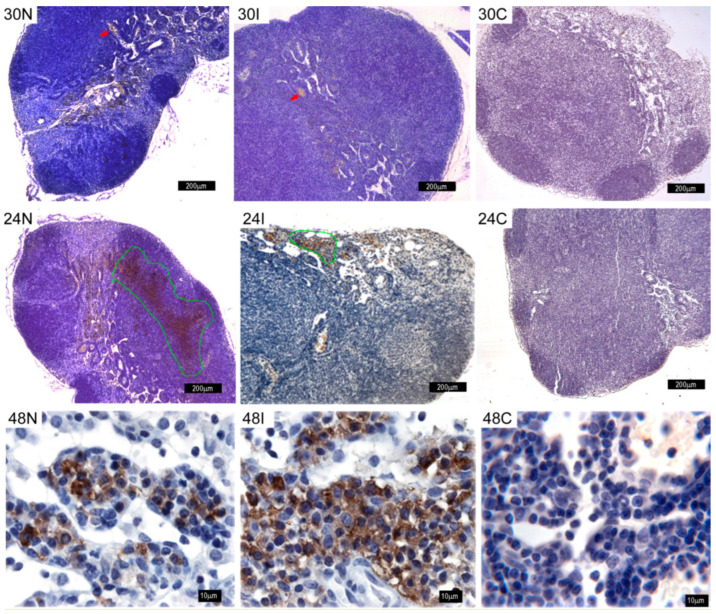
TNF-α staining in mandibular lymph nodes after Ph. duboscqi bites. TNF-α staining in mandibular lymph nodes after non-infected at 30 min, 24 h, and 48 h (30N, 24N, and 48N); *Leishmania*-infected sand fly bites (30I, 24I, and 48I); or in controls not exposed to sand fly bites (30C, 24C, and 48C). Red arrows indicate blood vessels of the medullary cords. Green outline shows TNF-α stain diffusely spread in the paracortical area of the lymph node. Brown staining (peroxidase) shows TNF-α concentrated within mononuclear cells (possibly lymphoid cells) of medullary cords, as well as diffusely spread within the lymphoid tissue. Bars = 10 μm in 48N, 48I, and 48C. Bars = 200 μm in 30N, 30I, 30C, 24N, 24I, and 24C. Representative images of each experimental condition from two independent experiments are shown; n = 4.

TNF-α release was also analyzed in the mandibular draining lymph nodes. A time-dependent accumulation of TNF-α was evidenced after infected and non-infected sand fly bites, which was absent in controls (Figure 11). At 30 min after infected and non-infected sand fly bites, initial TNF-α staining was detected preferentially in the blood vessels of the medullary cords of mandibular lymph nodes (Figure 11, red arrows). At 24 h, an enhanced TNF-α stain was diffusely spread in the paracortical area of the lymph node in infected and non-infected sand fly bites (Figure 11, green outline). It is noteworthy that at 48h after sand fly bites, a dramatic increase of the TNF-α stain was observed in infected and non-infected sand fly bites. Controls not exposed to sand fly bites or parasites did not show TNF-α staining in lymph nodes at all time points investigated.

## 4. Discussion

To the best of our knowledge, there are no studies on the role of MC during the initial acute inflammation that occurs after natural *Leishmania major* infections. In our current model, we studied the MC response to *L. major*-infected and non-infected *Ph. duboscqi* sand fly bites during the first 48 h after the bites. Our results show that sand fly bites lead to an increase in MC numbers, observed after 30 min and up to 48 h. Even though pressure exerted by application of empty vials caused an initial transient increase in mast cell numbers, these returned to basal levels after 24 h. In tissues exposed to infected and non-infected sand fly bites, MC increased in number continuously, reaching similar levels after 24 h. However, after this time point, MC in tissues exposed to infected bites continued to increase, reaching significantly higher levels as compared to non-infected bites at 48 h. Thus, the initial surge of MC numbers is more related to sand fly bites, whereas the later increase is potentially mediated by components of the infectious inoculum. It is not clear if the enhanced numbers are related to their migration from blood or from other tissue compartments.

Our data also show that MC degranulate almost immediately after sand fly bites, showing tryptase, histamine and TNF-α staining. After 6 h, many MC showed presence of different sized granules in the cell cytoplasm, suggesting a possible “re-granulation”. This observation leads us to propose that sand fly bites trigger tryptase and histamine production in MC at an early time point, and that these cells possibly continue producing these mediators by “de novo synthesis” for several hours. This is in accordance with the literature, where early “de novo synthesis” of different inflammatory mediators has been reported in MC activated with substance P and CD30 or after crosslinking of Fc**ε**RI, leading to their synthesis of eicosanoids (cysteinyl leukotrienes), cytokines (TNF-α), and chemokines [29,30,31]. Alternately, the presence of MC with granules after 6 h could also be a consequence of MC recruitment from surrounding tissues.

Though the number of MC was significantly higher after infected compared to non-infected sand fly bites, the staining for TNF-α, histamine, and tryptase was equally intense throughout the 48 h. Thus, the release of these proinflammatory mediators by MC is most likely related to the damage induced by the proboscis of the insect in addition to insect derived factors and not to the infectious inoculum.

Catalytically inactive histamine and tryptase are stored in the secretory granules of MC and their relatively higher secretion has been reported during acute inflammatory processes [1,32]. To the best of our knowledge, this is the first time that tryptase is demonstrated after a sand fly bite. The strong tryptase stain observed on endothelial cells of blood vessels after sand fly bites suggests that it may participate in vasodilatation and vascular congestion [33,34].

Histamine, another inflammatory mediator was evidenced after sand fly bites. Histamine is considered an immunomodulator of the innate and adaptive immune responses, generally leading to immunosuppression and favoring a Th2 response [35,36]. Thus, intense histamine stain evidenced in MC granules and surrounding tissues after sand fly bites possibly favors infection by the *Leishmania* parasite.

A further inflammatory impact caused by sand fly bites is the release of TNF-α found in MC granules to surrounding tissues. Although TNF-α has been associated with host protection in leishmaniasis due to induction of iNOS in mononuclear phagocytes in association with IFN-γ [37], it has also been related to disease severity and enhanced lesion sizes [38,39,40]. In contrast, in resistant C57BL/6 mice, TNF-α release by MC has been proposed to orchestrate the recruitment of neutrophils and macrophages [3,17]. The enhanced presence of TNF-α in MC after sand fly bites possibly has consequences relevant to disease development since it promotes an early neutrophilic recruitment, both to the site of bite, as well as to the mandibular draining lymph nodes. Although neutrophils initiate the sterilization response and tissue repair, they have also been shown to provide a temporal haven for the parasites, sheltering them from the deleterious effect of complement activation by acting as “Trojan horses” [22,23]. Our study shows that an important recruitment of neutrophils to tissues, as well as to the mandibular draining lymph nodes, occurs at 30 min after sand fly bites and persists throughout the 48 h of the study period. In contrast, controls only exposed to empty vials also showed a transient neutrophil infiltration, which progressively subsides reaching basal levels after 48 h. This transient increase seems nonspecific and is possibly related to the pressure exerted by application of the vials to mouse ears. It is noteworthy, that at 48 h, bites of non-infected sand flies induced higher number of neutrophils, as compared to Leishmania-infected sand fly bites. Our data support a study reporting the differential kinetics of neutrophil recruitment after infected and non-infected sand fly bites determined by flow cytometry [18] and contrasts with a study done with two-photon intravital microscopy, which showed an equally massive neutrophilic recruitment after infected and non-infected sand fly bites [22]. These discrepancies may be related to the use of different approaches. More studies are needed to determine the complete course of neutrophil infiltration into bite sites of infected and non-infected sand flies to identify parasite and sand fly mediators responsible for neutrophil recruitment. Recent studies have already identified a sand fly salivary protein with neutrophil chemotactic activity [41].

It is noteworthy, that neutrophils at the bite site also showed an intense TNF-α stain and, to the best of our knowledge, this is the first in vivo demonstration of TNF-α in neutrophils after infected and non-infected sand fly bites. In addition to the observed increase in the number of neutrophils in the tissue after sand fly bites, neutrophils also enter the paracortex and medullary cords of mandibular draining lymph nodes as early as 30 min and are present up to 48 h after a bite. It is not clear whether neutrophils also contribute to the enhanced TNF-α stain in the draining lymph node, since the intense stain was seen mostly in other mononuclear cells (possibly lymphoid cells). This is in accordance with the literature, where neutrophils have been shown to traffic to lymph nodes early after infection with pathogens such as *L. major*, *Mycobacterium bovis*, and *Toxoplasma gondii,* to interact with pathogens that egress from host cells [42,43,44,45]. During L. major infection, a transient neutrophilic infiltration into the cortex and subcapsular space of lymph nodes was reported during the first 24 h after infection [42]. Our study now shows that neutrophils transmigrate from HEVs to the medullary cords and the paracortex, accompanied by high cellularity in the subcapsular sinus, after sand fly bites. Of note, neutrophils have been observed in lymph nodes 16 h or less after intradermal injection of Leishmania major, and their possible role in the draining lymph nodes has been associated with the development of a Th2 response in the susceptible BALB/c model, since their absence led to a protective effect that seemed to be dependent on IL-12 [42].

The heavy neutrophilic infiltration into the draining lymph node is probably due to components associated to the sand fly, since a comparable response was observed after infected and non-infected bites. Neutrophils located in the lymph nodes could orchestrate the development of a strong immunity against components of the sand fly inoculum. The localization of neutrophils in the lymph nodes surely represents an advantage for the host; yet, it also represents an interesting evasion strategy for *Leishmania*, since the parasites entering the lymphatic drainage from infection sites can take advantage of these neutrophils as temporary shelters [22]. Leishmania parasites have been reported in lymph nodes from 16 to 24 h of experimental infections with *L. major* [39,42]. The presence of neutrophils in lymph nodes for at least 48 h possibly enables Leishmania to travel to distant sites after entering the blood circulation [46].

The limits of the current project are defined by the fact the we only focused on histological aspects, yet our results now provide evidence on the first events that occur after the natural transmission by vector bites, a topic where very limited data exist. The visual evidence of these initial events contributes to understand how the parasite takes advantage of the innate immune of the host generated by sand fly components for its successful establishment. Future molecular studies are needed to obtain more detailed information on the regulatory events involved.

## 5. Conclusions

In conclusion, this natural model of *Leishmania major* transmission by infected *Phlebotomus duboscqi* sand flies sheds new light on the early kinetics and response of MC during sand fly bites: they rapidly increase in number and show a massive release of granules containing potent inflammatory mediators. As such, MC participate in shaping the early host immune response after sand fly bites. The parasite can take advantage of these proinflammatory events inflicted upon the host by the sand fly bite that promote neutrophil recruitment, allowing the parasite to hide “undetected” inside neutrophils before an effective adaptive immune response is fully established. These finely tuned evasion mechanisms exerted upon the immune system of the host by the sand fly bite must be considered when designing therapeutic or preventive strategies for the control of leishmaniasis.

## Data Availability

Data contained within the article are available upon request.

## Supplementary Materials

The following supporting information can be downloaded at: https://www.mdpi.com/article/10.3390/pathogens12020207/s1: Supplemental Figure S1. Immunostaining of neutrophils for TNF-alpha in dermis of mice experimentally inoculated with Leishmania.
