# Supplementary material for: Leishmania major-Infected Phlebotomus duboscqi Sand Fly Bites Enhance Mast Cell Degranulation
Source: Pathogens. 2023 Jan 28;12(2):207. doi: 10.3390/pathogens12020207 (PMC9960273; doi:10.3390/pathogens12020207)
Supplement: Supplementary file 1 [file pathogens-12-00207-s001.zip › pathogens-2089586-supplementary.pdf]

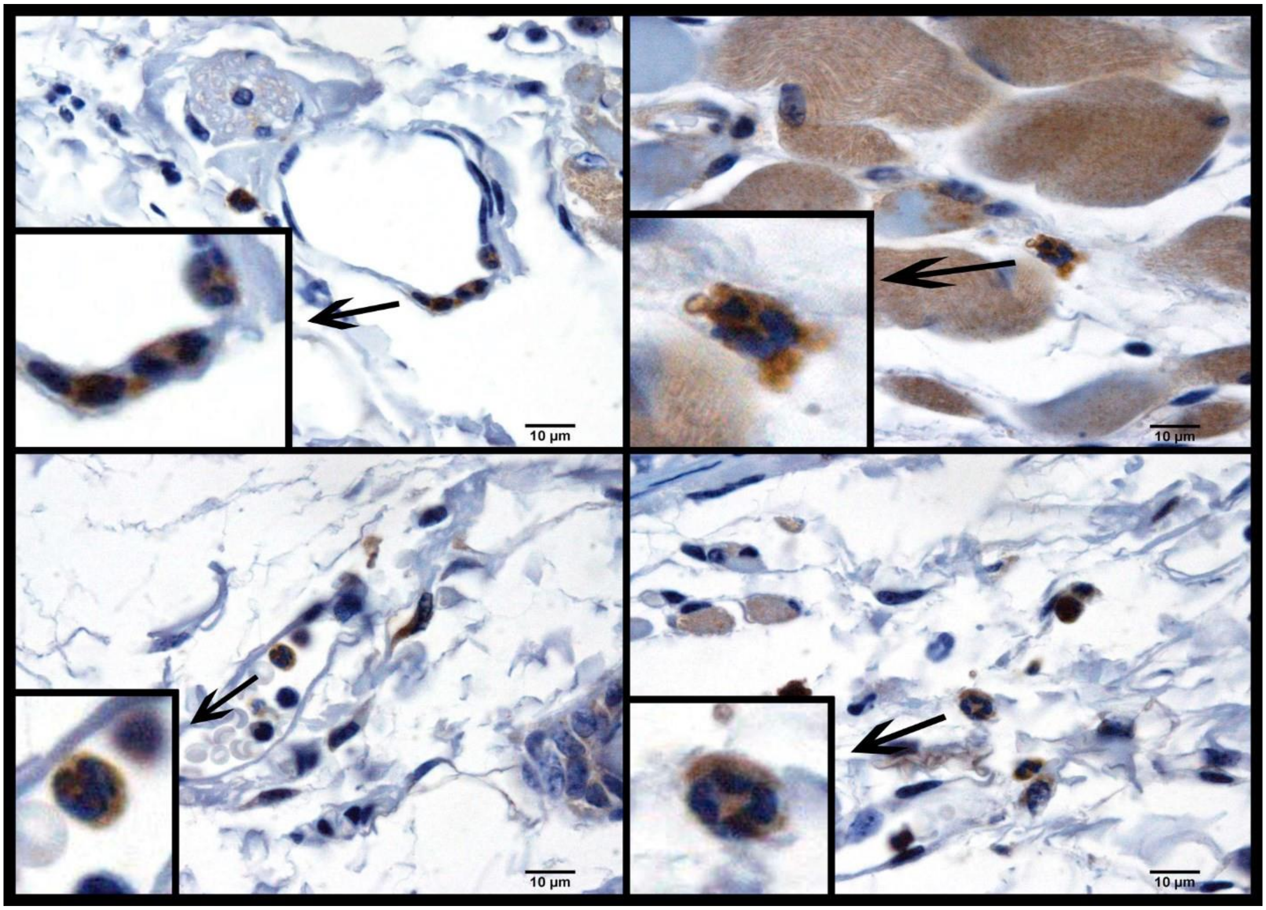

Supplemental Figure S1. Immunostaining of neutrophils for TNF-alpha in dermis of mice experimentally inoculated with *Leishmania.* Venules contain circulating, marginating or endothelium-adhered TNF-alfa-positive neutrophils, recognizable by 2 to 3 lobes of the nucleus (left panels). Extravascular neutrophils among muscle cells and in the connective tissue show cytoplasmic immunostaining for TNF-alfa (right panels). Arrows represent the enlarged image of the cells showing brown staining of TNF-alpha.
